# Epigenetic orchestration of sugar signaling in plant development and stress adaptation

**DOI:** 10.1093/hr/uhag109

**Published:** 2026-04-02

**Authors:** Ping Zhao, Yi Liu, Javeria Abid, Xingtan Zhang, Noor-ul Ain

**Affiliations:** National Key Laboratory for Tropical Crop Breeding, Shenzhen Branch, Guangdong Laboratory for Lingnan Modern Agriculture, Genome Analysis Laboratory of the Ministry of Agriculture, Agricultural Genomics Institute at Shenzhen, Chinese Academy of Agricultural Sciences, Shenzhen, Guangdong 518120, China; National Key Laboratory for Tropical Crop Breeding, Shenzhen Branch, Guangdong Laboratory for Lingnan Modern Agriculture, Genome Analysis Laboratory of the Ministry of Agriculture, Agricultural Genomics Institute at Shenzhen, Chinese Academy of Agricultural Sciences, Shenzhen, Guangdong 518120, China; National Key Laboratory for Tropical Crop Breeding, Shenzhen Branch, Guangdong Laboratory for Lingnan Modern Agriculture, Genome Analysis Laboratory of the Ministry of Agriculture, Agricultural Genomics Institute at Shenzhen, Chinese Academy of Agricultural Sciences, Shenzhen, Guangdong 518120, China; National Key Laboratory for Tropical Crop Breeding, Shenzhen Branch, Guangdong Laboratory for Lingnan Modern Agriculture, Genome Analysis Laboratory of the Ministry of Agriculture, Agricultural Genomics Institute at Shenzhen, Chinese Academy of Agricultural Sciences, Shenzhen, Guangdong 518120, China; National Key Laboratory for Tropical Crop Breeding, Shenzhen Branch, Guangdong Laboratory for Lingnan Modern Agriculture, Genome Analysis Laboratory of the Ministry of Agriculture, Agricultural Genomics Institute at Shenzhen, Chinese Academy of Agricultural Sciences, Shenzhen, Guangdong 518120, China

## Abstract

Sugars are not only metabolites but also signals that gate plant growth, fruit ripening, and stress responses. Carbon-sensing pathways (HEXOKINASE 1, TOR-SnRK1, and the sucrose Trehalose-6-phosphate pathway) reprogram gene expression by engaging DNA methylation, histone modification, chromatin remodeling, regulatory RNAs, and RNA modifications. Cutting-edge quantitative epigenomic and epitranscriptomic methods, integrated with novel bioinformatics tools, are driving unprecedented resolution in epigenetic landscape analysis. Benefitting from state-of-the-art technologies, we summarize epigenetic control points for pigmentation, softening, sugar–acid balance, and stress acclimation (e.g. *SlDML2*, *PH5*, RdDM-ABA modules). This synthesis underscores that decoding the sugar–epigenome interplay is key to understanding plant phenotypic plasticity and improving crop performance. Finally, we explore how leveraging epigenetic regulation via CRISPR-based epigenome editing, transgenerational inheritance patterns, and epigenomic biomarker-based (epibreeding) approaches holds promise for improving crop resilience and productivity.

## Introduction

In the context of plant biology, the interplay between genetic predisposition and environmental factors (G × E) is paramount in determining phenotypic outcomes under ever-changing conditions. Beyond DNA, epigenetics involves passing heritable changes in gene function, communicating with external environmental signals, which can induce stable, yet sometimes reversible, modifications in gene expression. Key epigenetic mechanisms, including DNA methylation, post-translational histone modifications, chromatin remodeling complexes, and regulatory noncoding RNAs, collectively orchestrate the spatial and temporal dynamics of the plant transcriptome and development [1]. This molecular versatility underlies the remarkable plasticity of sessile organisms, enabling them to rapidly acclimate and adapt to fluctuating abiotic and biotic stresses over the long term. A central question in plant biology is how plants recruit these epigenetic processes in synchronization with growth and external signals to fine-tune developmental programs and also pass this behavior to other generations.

Plants are obliged to run an internal carbon economy from juvenility to senescence. Apart from their roles as structural and metabolic pillars of carbon, sugar molecules operate as signaling entities, integrating information on nutrient status, developmental changes, stress onset, circadian rhythm, ecological and climatic fluctuations, triggering epigenetic reprogramming that leads to phenotypic plasticity [2]. This remodeling of physiological or developmental adaptations is transient, or plants may transmit trans-generationally without modifying genomic sequences, leading to a phenomenon called epimutation, which arises from epialleles. These signatures of epigenetic mutations may vary in terms of genome-wide contiguity, which is more prevalent in complex genomes with more transposable elements (TEs) in flowering plants [3]. Similarly, differential profiles of metabolic and sugar-signaling pathways along spatiotemporal spectrums within similar species or varieties relate to epiallele mutations [4]. Plants have improved sugar signaling pathways during evolutionary trajectories, using them as ligands in molecular signaling networks, resulting in appropriate cellular responses [5]. Sugar signaling relies on an intrinsic sensory system involving various regulatory networks to manage plant growth and development. This sensory system involves sensors or transporters that respond to carbohydrate concentration, the carbon-to-nitrogen (C/N) ratio, and metabolic flux. There are many examples of CRISPR Cas9-based interventions exploiting methylation and demethylation-based epigenetic editing, reshaping many agronomic traits [6, 7]. These advances underscore the potential of ‘epibreeding’ to bypass genetic erosion and accelerate the development of stress-adapted cultivars.

The understanding of sugar's biology has matured into a mechanistically rich field, yet the regulatory logic connecting carbon status to durable gene-expression programs remains fragmented. What is still missing is a coherent, causal, and testable framework that explains when sugars simply change transcript levels transiently versus when sugar status reconfigures chromatin competence in ways that bias development, fruit ripening, and stress adaptation over meaningful timescales. The past few years, however, have delivered precisely the kind of mechanistic coupling evidence that enables a more rigorous synthesis.

We synthesize evidence across developmental transitions, fruit quality traits, and stress responses, with special attention to horticultural and polyploid crops where (i) sugar partitioning is extreme, (ii) allele or subgenome-aware epigenomic variation is biologically and agronomically consequential, leading to variable resilience or agronomic traits, and propose experimentally tractable models that can be falsified. Before contextualizing sugar mechanisms, we first provide a brief overview of key biological and technological advancements in epigenetics to establish a foundation for the readers.

## Advances in epigenomics: tools, discoveries, and ecological insights

### The evolution of epigenomic understanding: past to present

Early insights into epigenetic regulation emerged from studies of paramutation, a phenomenon in which one allele transcriptionally silences its homologous counterpart, leading to transgenerational epigenetic inheritance. This silencing is maintained by small interfering RNAs (siRNAs) and the RNA-directed DNA methylation (RdDM) pathway [8, 9]. Vernalization in *Arabidopsis* epigenetically switches *FLOWERING LOCUS C* (*FLC)* from an active state, marked by H3K4me3 and H3K36me3, in warm seasons to a silenced state, marked by H3K27me3, after prolonged cold. This process enables spring flowering and resetting each generation, illustrating inheritance based on chromatin and RNA interference (RNAi) with applications for breeding and resilience. These foundational studies demonstrate RNAi- and chromatin-driven epigenetic inheritance in plants, enabling nutrient enhancement, climate resilience, and synthetic biology applications by bridging genome–environment–evolution dynamics for next-generation breeding.

### Advancing quantification methods from the epigenome to the epitranscriptome in plants

With high-throughput sequencing, epigenetics has shifted from a descriptive to a quantitative approach, enabling the genome-wide measurement of regulatory layers. Epigenomic studies decipher chromatin accessibility and transcriptional activity through the analysis of DNA methylation, histone modifications, and chromatin remodeling. In contrast, the epitranscriptome encompasses chemical modifications on RNA molecules—most notably N6-methyladenosine (m^6^A), 5-methylcytosine (m^5^C), and pseudouridine (Ψ)—which critically influence RNA splicing, stability, nuclear export, and translation efficiency [10]. For chromatin-level regulation, key assays include bisulfite sequencing (BS-seq) for DNA methylation profiling, chromatin immunoprecipitation followed by sequencing (ChIP-seq) for detecting histone modifications, and the assay for transposase-accessible chromatin using sequencing (ATAC-seq), enabling comparative analyses across tissues, genotypes, and environments.

Epitranscriptome mapping initially relied on m^6^A, which is primarily detected using methylated RNA immunoprecipitation sequencing (MeRIP-seq), which provides transcriptome-wide enrichment but typically lacks single-nucleotide resolution. To address this limitation, high-resolution techniques, such as MAZTER-seq, m^6^A-CLIP, miCLIP, and DART-seq, infer reverse transcription stalling, crosslinking-induced mutations, or enzymatic editing to achieve greater positional accuracy [11, 12]. In plant systems, technical challenges arise from factors such as low RNA abundance, high tissue heterogeneity, and RNA degradation. Recent advances in direct RNA sequencing technologies, particularly Oxford Nanopore's platform, have enabled the simultaneous detection of multiple RNA modifications without requiring reverse transcription, representing a promising frontier in quantitative epitranscriptome research [11]. For other modifications, such as m^5^C and Ψ, detection methods include RNA BS-seq, Aza-IP, Pseudo-seq, and CeU-seq [13]. Each method offers specific strengths in terms of sensitivity, specificity, and throughput, while also facing technical challenges such as background noise, heterozygosity, intricate polyploid genomes, incomplete chemical conversion, and false positives that require attention [12, 14]. Considering the expanded and diverse epigenomic and epitranscriptomic datasets, analysis is increasingly limited by signal-to-noise, confounding by expression/coverage, and isoform ambiguity, requiring robust statistical and machine learning (ML) frameworks that incorporate both m^6^A-IP/MeRIP-seq and Nanopore direct RNA sequencing. ML increasingly complements inference by enabling site prediction, stoichiometry estimation, and cross-species transfer under sparse labels; key plant-focused predictors and differential-modification workflows are compiled in Table 1.

**Table 1:** Computational and machine-learning tools for plant epigenetic and epitranscriptomic inference.

Tool	Application	References
EpiDiverse EWAS Pipeline	Links DNA methylation patterns to genotype × phenotype variation in non-model plants using bisulfite sequencing and SNP data.	[15]
MethylC-analyzer	Analyzes plant methylomes using BS-Seq and EM-Seq; identifies CG/CHG/CHH methylation changes and generates visual outputs.	[16]
MethylScore	Uses ML to identify differentially methylated regions across large plant populations for genotype–phenotype studies.	[17]
sounDMR	Performs population-level DMR analysis using Nanopore data; detects within-group epigenetic variability in plants.	[18]
SMEP	Deep learning framework to predict multiple epigenetic marks, including 5mC, 6mA, m^6^A, histone modifications from genomic sequences in crops like rice and maize.	[19]
DeepSignal-plant	Detects genome-wide 5mC methylation in all plant contexts (CG, CHG, CHH) using Nanopore reads; correlates methylation with phenotype.	[20]
PEA-m^6^A (Plant epitranscriptome analysis—m^6^A)	Cross-species m^6^A site prediction using transfer learning; works with small sample sizes.	[21]
PEA-m^5^C (Plant epitranscriptome analysis—m^5^C)	Predicts transcriptome-wide m^5^C sites; analyzes positional distribution, such as regions near start codons.	[22]
deepEA (deep epitranscriptome analysis)	All-in-one epitranscriptome analysis platform; supports peak calling, differential methylation, visualization, and even ML-based m^6^A prediction.	[23]
RADAR (RNA methylAtion differential analysis in *R*)	Identifies loci with differential m^6^A levels across conditions/groups; accounts for input expression and study design.	[24]
xPore	Quantifies per-site modification rates and identifies differential m^6^A in ONT DRS data without matched controls.	[25]
CHEUI (CH3 estimation using ionic current)	Simultaneous detection of m^6^A and m^5^C in individual ONT reads; identifies co-occurring modifications; includes a differential-testing module.	[26]

### The ‘resolution’ revolution and single-cell epigenomics

Single-cell epigenomics involves the high-resolution profiling of transcriptomics, DNA methylation, chromatin accessibility, histone modifications, and three-dimensional (3D) genomic architecture at the single-cell level, minimizing bulk-level quantification and heterogeneity. However, in plant cells, cell walls pose challenges; single-nucleus RNA sequencing (snRNA-seq) serves as a robust alternative [27], with nuclear RNA capturing rapid transcriptional changes [28]. Despite slower progress in plants due to genomic complexity, bisulfite sequencing of rare input fragments sequencing (BRIF-seq) revealed extensive DNA methylation reprogramming during maize male gametophyte development [29]. Chromatin accessibility, detected by single-cell assay for transposase-accessible chromatin sequencing (scATAC-seq) [30], is often integrated with transcriptomics. Emerging plant applications include studies of peanut fruit development [31] and soybean cell types [32]. Similarly, histone modifications and 3D genome architecture profiling were developed recently using single-nucleus cleavage under targets and tagmentation sequencing (snCUT&Tag), profiling H3K4me3 in rice seedlings [33], and single-cell chromatin conformation capture (scHi-C) in rice gametes and zygotes [34]. The underutilization of these tools in plants is due to extended repetitive genomes and technical barriers, such as protoplasting, while polyploidy limits the resolution.

The emergence of contemporary epigenomic and epitranscriptomic data necessitates the development of sophisticated bioinformatics tools. The analysis of epitranscriptome data depends on specialized bioinformatics tools tailored for the identification and quantification of RNA modifications. For m^6^A, software tools such as m6AViewer, exomePeak, and MeTPeak, are widely used for peak calling and statistical inference [12]. Additionally, pipelines like ModTect and Nanocompore integrate alignment algorithms and statistical models to detect modification signals, often incorporating ML, signal decomposition, or probabilistic inference to distinguish true biological modifications from sequencing noise. Despite these advances, substantial computational challenges persist. RNA modification signals are typically weak, context-dependent, and sparsely distributed, while technical variability and limited cross-study reproducibility hinder robust detection. Moreover, factors such as transcript isoform complexity, isotope substitutions, and the lack of high-fidelity datasets, further complicate analysis [14, 35]. Developing high-sensitivity, cross-platform-compatible algorithms capable of resolving dynamic stoichiometry and capturing spatio-temporal variation remains a major priority in advancing epigenome and epitranscriptome research [35].

### The premise: sugars as messages, epigenome as decoder

**Figure 1:**
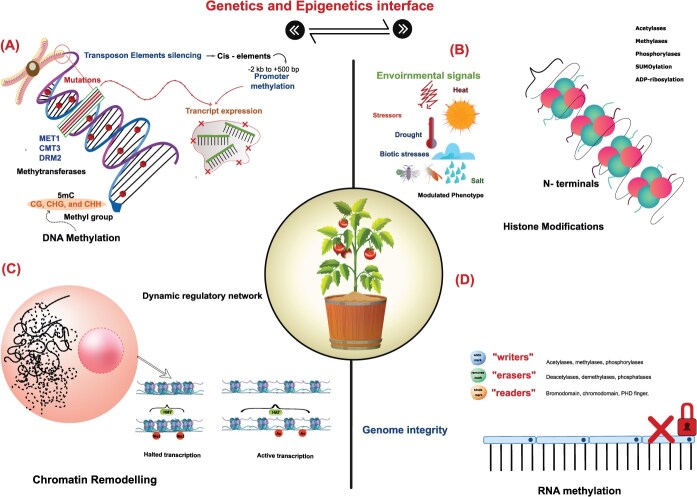
Synchronization of genetics and epigenetics for regulating environmental cues to modulate phenotype. (A) Dynamic CG/CHG/CHH methylation by METHYLTRANSFERASE 1 (MET1), CHROMOMETHYLASE 3 (CMT3), and DOMAINS REARRANGED METHYLTRANSFERASE 2 (DRM2) modulates promoter accessibility, which leads to varied molecular dynamics that orchestrate ripening. (B) Stress signals induce histone modifications via ‘writers’, ‘erasers’, and ‘readers’. (C) Chromatin remodeling dynamically alters nucleosome organization to activate or repress transcription. (D) RNA methylation adds an epitranscriptomic layer, contributing to gene regulation and genome integrity.

In recent years, empirical insights into the dynamic interactions between the genome and the environment have helped decipher the plasticity responses to environmental stress in plants. Moving beyond the static genetic code, epigenetic regulation operates through multilayered processes spanning whole-genome bisulfite sequencing (WGBS), which has revealed differentially methylated regions (DMRs) near sugar metabolism genes, such as sucrose-phosphate synthase (*SPS*) and fructose-1,6-bisphosphatase (*FBPase*). In parallel, recent nanopore long-read methylation sequencing has enabled subgenome-level 5mC profiling to uncover the regulation of SWEET sugar transporters. Furthermore, ChIP-seq analyses define activation and repression states mediated by histone marks, while ATAC-seq links open chromatin landscapes to fruit sugar-related programs. Additionally, m^6^A RNA epitranscriptomic marks shape the transcript stability of stress and sugar signaling pathways [12]. The following realms provide a systematic overview of how these multilayered epigenetic regulatory machineries coordinate to form a complex network that extends from the genome to the transcriptome and modulates the phenotype (Fig. 1A–D). Accordingly, Table 2 summarizes the profiling and editing methods, including CRISPR/dCas9, used to connect these epigenetic layers with sugar-linked phenotypes.

**Table 2:** Experimental epigenomic tools for profiling and editing epigenetic and epitranscriptomic modifications in plants.

Tool	Crop	Application	References
WGBS	Sugarcane (*Saccharum* spp*.*)	Genome-wide cytosine methylation mapping across leaf, root, rind, and pith. Identified DMRs and ‘methylation valleys’ in sugar metabolism genes, including *SPS* and *FBPase.*	[36]
ChIP-seq	Sorghum (*Sorghum bicolor*)	H3K4me3 and H3K27me3 profiling under low phosphorus in roots. Revealed histone methylation in phosphate-starvation response.	[37]
ATAC-seq	Tomato (*Solanum lycopersicum*)	Chromatin accessibility in normal vs. long-shelf-life fruits. Found activation of *TCP* TFs and sugar metabolism genes.	[38]
ATAC-seq + RNA-seq	Grapevine (*Vitis amurensis*)	Cold stress-induced TFs (*RAV1*, *CBF4*, *ERF104*) showed chromatin opening and transcriptional upregulation.	[39]
CRISPR-dCas9-SunTag-TET1	*Arabidopsis thaliana*	Targeted demethylation of *FWA*, *CACTA1* loci to reactivate silenced genes.	[40,41]
MSAP (methylation-sensitive AFLP)	Rapeseed (*Brassica rapa*)	Identified cold-induced methylation changes; ~41% loci altered post-treatment.	[42]
m^6^A-seq/MeRIP-seq	Maize (*Zea mays*), *A. thaliana*	RNA methylation profiles under salt and heat stress; regulation of sugar metabolism and stability.	[43,44]
Nanopore long-read methylation sequencing	Bread wheat (*Triticum aestivum*)	Subgenome-level 5mC profiling; regulation of SWEET sugar transporters and flowering genes, including *VRN1* and *FT*.	[45]
CRISPR-dCas9-MQ1	*A. thaliana*	CG-specific DNA methyltransferase activity for site-specific CG methylation at loci such as *FWA*, resulting in stable gene silencing.	[46]

### The sugar signaling network from the perspective of epigenetics

**Figure 2:**
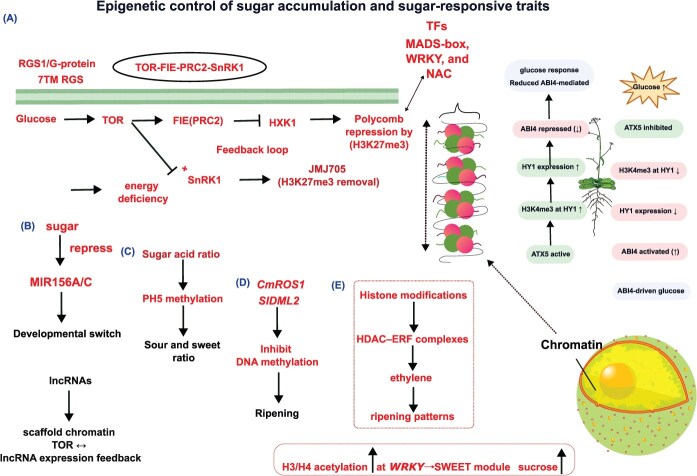
Epigenetic links between sugar signaling and development, flavor, and ripening. (A) Glucose sensing converges on a TOR-PRC2/*SnRK1* hub to tune H3K27me3 dynamics and sugar-responsive TF programs. (B) Sugar-regulated miRNAs and lncRNAs shape developmental transitions via chromatin feedback. (C) DNA methylation modulates sugar-acid balance. (D) DNA demethylation promotes ripening gene reprogramming. (E) Histone modifications integrate ethylene and TF control to pattern ripening and increase sucrose accumulation.

The perception of carbon status occurs through the direct nuclear regulatory activity of HEXOKINASE1 (HXK1), as well as extracellular sugar perception via plasma-membrane RGS1/G-protein circuitry, which serves as an indirect sensing mechanism through metabolic and energy relays [47]. These inputs converge on a small set of master hubs that reprogram transcription. Here, Target of rapamycin (TOR) integrates glucose-derived bioenergetic flux to promote cell-cycle competence, translation, and meristem activation, whereas SNF1-RELATED PROTEIN KINASE 1 (SnRK1) feeds back during starvation and prioritizes catabolism, restrains growth, and enhances stress resilience. A pivotal refinement of this framework is the sucrose Trehalose-6–phosphate (T6P) activity, which functions as a proxy for sucrose availability and restrains SnRK1 activity. This master knob balances growth and defense, linking carbohydrate supply to major transitions, such as early maturity (Fig. 2A).

This interactive signaling with the environment supports the theme that sugar signaling is inherently epigenetic, and carbon availability is tuned into chromatin states that are stable enough to impose developmental directionality, yet plastic enough to accommodate fluctuating environments. Critically, sugar signaling also interfaces with epigenetic machinery to stabilize long-term responses. A landmark advance showed that glucose-activated TOR can directly engage Polycomb repression by targeting FIE and polycomb repressive complex 2 (PRC2), reshaping the genome-wide H3K27me3 landscape. This leads to sugar sufficiency as a nutritional checkpoint that gates organogenesis and developmental timing [48]. Conversely, the energy-deficient arm is also chromatin-embedded. For instance, in rice, SnRK1 promotes tolerance to energy limitation through activation of the H3K27me3 demethylase JMJ705, revealing a direct mechanistic bridge from energy sensing to reversible removal of repressive marks [49]. Collectively, these discoveries support a unifying model in which the TOR–FIE–PRC2–SnRK1 axis not only changes transcript levels through classical signaling cascades but also reconfigures chromatin to determine which diverse gene networks can be rapidly induced or durably constrained.

### Sugar-mediated chromatin remodeling

Sugar signaling, chromatin remodelers, and transcriptional interplay, along with histone modifications, dynamically regulate sugar-responsive gene expression. In *Arabidopsis*, ARABIDOPSIS TRITHORAX-RELATED 5 (*ATX5*) trimethylates H3K4 at HYPOCOTYL 1 (*HY1*), repressing ABSCISIC ACID INSENSITIVE 4 (*ABI4*); glucose inhibits *ATX5*, lowers H3K4me3, and activates the ATX5-HY1-ABI4 module in glucose signaling. In rice, the SUCROSE NON-FERMENTING 2 (*SNF2*) remodeler LATERAL FLORET 2 (LF2) directs H3K27me3 deposition to modulate genes governing lateral spikelet development and organ identity [50]. Additionally, sugar-responsive TFs *WRKY18/53* bind W-box motifs, recruit the histone acetyltransferase HAC1 to deposit H3K27ac, and activate sugar-inducible genes [44]. In apple, differential promoter methylation of key genes related to sucrose metabolism, hormone biosynthesis, and signaling, including *ACS*, *ACO*, *ZEP*, and *NCED*, as well as transcription factors such as MADS-box, WRKY, and NAC, alters hormonal crosstalk and sugar fluxes, thereby modulating fruit ripening and quality formation [51].

In the context of sugar signaling, HXK1 forms a complex with the PRC2 core components SWINGER (SWN) and CURLY LEAF (CLF)*,* targeting specific loci for H3K27me3 deposition and repressing the transcription of glucose-responsive genes [52]. The histone acetyltransferase HAC1 catalyzes H3K27ac at sugar-responsive genes to promote transcriptional activation, and its loss-of-function mutants exhibit reduced sugar sensitivity and impaired fertility [53]. Under sugar starvation, a rapid loss of H3K9 methylation is observed, correlating with cell cycle arrest and the activation of cell differentiation [54]. Conversely, sufficient sugar availability induces histone H3 acetylation (H3KAc) at promoters, such as that of FCS-LIKE ZINC FINGER 8 (*FLZ8*), promoting its expression and activating the *TOR* signaling pathway, which establishes a feedback loop that balances growth and stress responses [55].

Histone modifications also modulate fruit ripening through regulation of carotenoid biosynthesis, sugar metabolism, cell wall degradation, and hormone signaling pathways. In this regard, histone acetylation and H3K4me3 promote ripening, while H3K27me3 exerts a repressive effect [56]. In light-mediated shade avoidance responses, the transcription factor PHYTOCHROME INTERACTING FACTOR 7 (PIF7) recruits methylation readers MORF RELATED GENE 1/2 (MRG1/MRG2), which recognize H3K4me3/H3K36me3 and promote H3/H4 acetylation, thereby activating genes, such as *YUCCA8*, *IAA19*, and *PRE1*, involved in auxin and brassinosteroid signaling [57]. Additionally, phytochrome B (phyB) can repress growth-related gene expression by interacting with the PRC2 subunit VIN3-LIKE 1 (VIL1) to promote H3K27me3 deposition and chromatin condensation [58]. These examples collectively underscore how histone methylation and acetylation integrate with remodelers and TFs to drive dynamic, sugar-responsive chromatin regulation.

Despite recent advancements, current models often treat histone marks as static, overlooking their combinatorial and context-specific functions. Recruitment specificity of modifiers like PRC2 by HXK1 or phyB is still unclear, and the dynamics of metabolic feedback loops, such as sugar-induced acetylation or starvation-triggered demethylation, remain poorly understood. Integration of simultaneous signals, like sugar and light, is also not well characterized at the chromatin level. Moreover, noncanonical histone modifications, such as ubiquitination and SUMOylation, are largely neglected in plants in this context. Future studies should utilize time-resolved, multiplexed epigenomics to capture the dynamic interplay of marks, identify locus-specific recruitment factors, quantify metabolite–chromatin feedback, and develop tools to explore underrepresented modifications, ultimately moving toward predictive models of epigenetic regulation.

### Post-transcriptional regulation of sugar homeostasis

In addition to chromatin-mediated controls of sugar-responsive transcription, post-transcriptional regulation contributes to sugar homeostasis by shaping transcript abundance and developmental timing outputs downstream of sugar availability. The expression of microRNAs (miRNAs), such as miR156, is closely associated with plant developmental age and is modulated by sugar signals; sugar represses *MIR156A/C* expression, thereby facilitating the transition from juvenile to adult vegetative phases [59, 60]. Notably, miR156 is expressed at lower levels in actively dividing tissues and accumulates in nondividing zones. This suggests a coupling between its expression and cell proliferation status, highlighting its role as a key epigenetic regulator in developmental programming [61]. Another stress-responsive miRNA, miR398, regulates the expression of copper/zinc superoxide dismutases (CSDs) and is essential for managing oxidative stress under sugar signaling. By maintaining redox homeostasis, miR398 indirectly influences sugar metabolic balance, thereby contributing to plant stress resilience [62, 63]. At the chromatin level, long noncoding RNAs (lncRNAs) can modulate gene expression by recruiting or inhibiting the binding of chromatin-modifying enzymes [64]. Post-transcriptionally, lncRNAs act as molecular sponges in competitive endogenous RNA (ceRNA) networks, sequestering miRNAs to relieve repression on their target mRNAs. This mechanism contributes to complex regulatory networks, especially under stress conditions [65]. Increasing evidence also highlights the emerging connection between lncRNAs and sugar metabolism. Therefore, TOR signaling pathway, a central regulator of growth and metabolism, modulates the expression of lncRNAs, which in turn affect gene transcription and stress adaptation [66] (Fig. 2B).

### m^6^A: rapid, condition-dependent tuning of RNA fate with chromatin crosstalk

The m^6^A modification is one of the most abundant and well-characterized epitranscriptomic modifications of mRNA in eukaryotes. Beyond direct regulation of mRNA fate, m^6^A modification also engages in intricate crosstalk with other epigenetic mechanisms, such as DNA methylation and histone modifications, thereby deepening its influence on gene expression regulation. For instance, m^6^A deposition on specific transcripts can affect the recruitment of chromatin modifiers, thereby forming a feedback loop between transcriptional and post-transcriptional regulation [67]. Furthermore, m^6^A often co-localizes with active histone marks such as H3K36me3, which is associated with open chromatin states and sustained transcriptional activity [68, 69]. Additionally, m^6^A modification can recruit specific protein complexes that influence the activity of DNA methyltransferases, thereby inducing locus-specific changes in DNA methylation and demonstrating the potential of RNA modifications to modulate chromatin architecture [70]. In *Arabidopsis*, m^6^A modification has been shown to participate in the regulation of lncRNA, particularly *COOLAIR*. Mechanistically, m^6^A facilitates the interaction of *COOLAIR* with FLOWERING CONTROL LOCUS A (FCA) and the 3′ RNA processing factor FY, thereby modulating the expression of *FLC* and influencing flowering time [71].

In sugar metabolism, m^6^A modulates the stability of transcripts encoding glycosidases, sugar transporters, and sugar-sensing factors, thus linking metabolic adjustment to stress adaptation [72]. In kiwifruit [73] and strawberry [74], m^6^A influences the expression of genes involved in organic acid and sugar metabolism, thereby modulating fruit flavor and quality. During fruit development, the m^6^A demethylase SlALKBH2 promotes tomato fruit ripening by stabilizing the mRNA of the DNA demethylase *SlDML2* [75, 76]. Collectively, these findings highlight that m^6^A is not only a rapid response mechanism to environmental change but also a critical regulatory hub in plant growth, development, and metabolic homeostasis. Studies have shown that, in sugarcane, m^6^A modifications accumulate notably in the 3′UTR during drought and other stress response [72]. These findings indicate that m^6^A, as an epitranscriptomic regulator, operates through highly condition-dependent mechanisms influenced by the type and duration of stress, as well as tissue specificity and species background [77].

## Subgenome dominance and DNA methylation dynamics: epigenomic drivers of fruit development and stress adaptation

**Figure 3:**
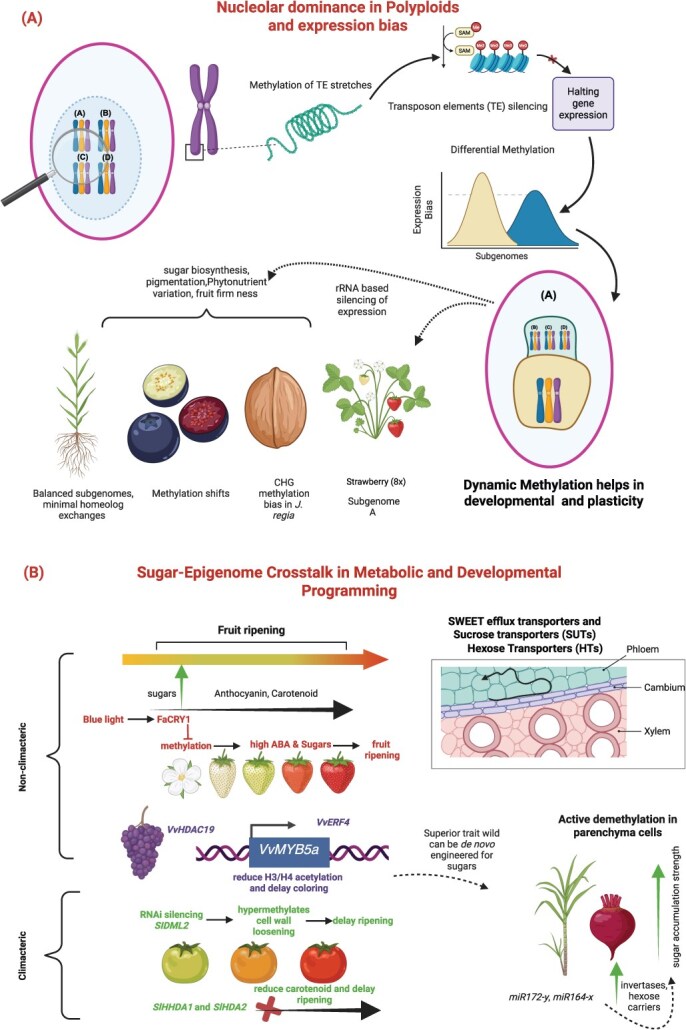
Epigenetic regulation of polyploid adaptation and sugar-driven fruit ripening. (A) Nucleolar dominance in polyploids: DNA methylation biases observed in subgenome expression, such as strawberry subgenome A, and stabilized TEs (B) Sugars recruit histone deacetylases (HDACs)/DNA methylases to control anthocyanin-mediated pigmentation, cell-wall-related softening, and ABA/ethylene crosstalk.

Recent advances in haplotype-resolved polyploid genomes and allele-aware epigenomics, including WGBS and long-read methylation profiling, enable investigation of how epigenetic regulation shapes fruit development, quality, and stress resilience in polyploid crops (Fig. 3A). This is important because many fruit crops are polyploid and clonally propagated, demanding stable performance across seasons as a primary breeding and production goal. Haplotype-resolved assemblies facilitate the exploration of subgenome expression, which is often accompanied by asymmetric regulation of repeats and TEs and associated with trait-relevant pathway deployment. More recently, a phased, gap-free octoploid strawberry genome explicitly linked genetic and epigenetic divergence among subgenomes and differential methylation patterns relevant to fruit biology, facilitating trait inference within the sugar-signaling epigenome [78, 79].

To get empirical insights into the epigenetic basis of expression dominance, recent studies have encompassed several polyploid species, most of which dictate neutral-to-non-neutral selection transitions and adaptive evolution [80–83]. A recent study integrated epigenomic and transcriptomic analyses of leaves and fruit skins, revealing how DNA methylation regulates subgenome dominance between two walnut species. Notably, genes exhibiting biased expression were enriched in pathways responding to external stimuli, whereas non-biased genes were associated with the assembly of signal transduction complexes [84].

DNA methylation contributes to promoting species diversity and enhancing environmental adaptability [85]. In strawberries, dynamic changes in DNA methylation are associated with stress responses, climate adaptation, and dormancy induction [86, 87], particularly closely linked to the genomic features of octoploid strawberries [88, 89]. Song and co-workers assembled and analyzed the genome of the octoploid ‘Benihoppe’ strawberry, revealing the dominant role of subgenome A in DNA methylation changes, which were found to be closely related to key traits in fruit development [90]. Subgenome A-biased genes were enriched in biological processes related to fruit development, with fruit maturation-related genes. In contrast, although subgenomes B, C, and D contain equivalent amounts of repetitive sequences, their methylation levels differ, particularly for TEs near genes. In *Brachypodium hybridum*, recurrent allotetraploidization events exhibit minimal homeologous exchanges (HEs) and balanced expression between subgenomes, indicating immediate genomic stability following polyploidization [91, 92]. Transposon density near genes did not drive dominance but was inherited from progenitors. These findings highlight the complex evolution and origin of allopolyploid genomes, tailored by epigenetic drivers. In the tetraploid blueberry (*Vaccinium corymbosum*), variations in phytonutrient pathways provided insights for improved fruit quality. Subgenome dominance occurs in blueberry, but expression shifts in fruit are linked to homoeologous structural variation [93]. Another study on similar species identified *VcTBL44* for firmness and β-alanine pathway methylation changes for climate adaptation, providing targets for future blueberry breeding [94].

Advances in epigenomic technologies reveal that subgenome dominance in methylation patterns in polyploids may be a dynamic driver of adaptation, yet causal mechanisms remain elusive. Comparative and functional methylome editing could disentangle adaptive from correlative patterns. Integrating structural variation mapping with epigenetic profiling offers a powerful route to link genome architecture for engineering against environmental diversity. This knowledge can be exploited for dissecting synthetic or neo-polyploids, proposed to be engineered with stoichiometrically balanced stress-response networks. Similarly, stress-induced patterns, for example, how alternate heat and cold spells induce the dynamics of the haploidy inducer *CENH3* with homeolog expression in polyploid berries, especially for sweetness, could resolve diverse research gaps. Rapid genome exchanges in synthetic polyploids reveal fast evolution, but timing in perennials is unknown; tracking in fast-cycling crops, exploiting epigenetic diversity, and integrating genomics with synthetic biology could unlock resilient, high-efficiency varieties and knowledge resources useful for perennials.

## Sugar-responsive epigenetic networks shaping fruit quality traits

Sugars in plants serve not only as metabolic currencies but also as signaling molecules that coordinate developmental programs and stress responses. In fleshy fruits, sugar signaling interfaces with chromatin regulators to fine-tune ripening traits, including pigmentation, softening, and aroma formation through DNA methylation and histone modification pathways (Fig. 3B). Here, we synthesize conserved and lineage-specific themes across fruit species, such as tomato, grape, strawberry, lemon, and melon, highlight causal epigenetic nodes responsive to sugar and hormone cues, and outline research directions that bridge metabolism with chromatin state dynamics [95–97]. Table 3 summarizes how epigenetic regulators, including DNA/RNA methylation and noncoding RNAs, directly influence sugar metabolism genes in diverse crops during development or stress responses.

**Table 3:** Epigenetic regulation of sugar metabolism in crop species

**Crop species**	**Epigenetic mechanism**	**Sugar metabolism genes/pathways affected**	**Developmental or stress context**	**Epigenetic effect on sugar accumulation**	**References**
**Tomato (*Solanum lycopersicum*)**	miRNA (*SlMIR164A*)	Sugar and organic acid metabolism (via *SlNAM2/3*)	Fruit ripening	*SlMIR164A* loss accelerates ripening and alters sugar/organic acid levels	[98]
**Grape *(Vitis vinifera*)**	DNA methylation	Brassinosteroid-related sugar partitioning genes	Berry development	Differential methylation of brassinosteroid pathway genes correlates with higher sugar (total soluble solids)	[99]
**Strawberry (*Fragaria ananassa*)**	RNA m^6^A demethylation (FvALKBH10B)	Sucrose, glucose, and fructose metabolism	Fruit ripening	Delayed ripening and reduced sucrose, glucose, and fructose levels	[100]
**Walnut (*Juglans regia*)**	miRNAs	Starch and sucrose metabolism pathway genes	Cold stress in seedlings	Cold stress alters miRNAs targeting starch/sucrose pathway genes, modulating sugar metabolism	[101]
**Sugar Beet (*Beta vulgaris*)**	DNA methylation	DNA methylation/demethylation genes	Cold stress in leaves	Cold induces genome-wide DNA hypomethylation (CHH) in sugar beet, reflecting stress adaptation	[102]
**Sugarcane (*Saccharum* spp.)**	DNA methylation	Sucrose synthesis genes (*SPS*, *FBP*ase) and TFs (WRKY, bZIP)	Tissue differentiation across leaf, rind, and pith	Tissue-specific methylation (DMRs/DMVs) overlaps sucrose synthesis genes (*SPS*, *FBPase*)	[36]
**Sweet orange (*Citrus sinensis*) **	Global DNA methylation	Lower demethylase expression in peel	Fruit ripening	Controls tissue-specific sugar accumulation and ripening	[103, 104]
**Apple (*Malus domestica*) **	DNA methylation of promoters	Sucrose metabolism genes, including *ACS*, *ACO*, *ZEP*, *NCED*; MADS-box, WRKY, NAC TFs	Fruit development and ripening	Alters sugar fluxes and hormone coordination for quality traits	[51]
**Arabidopsis (*Arabidopsis thaliana*) and Rice (*Oryza sativa*)**	DNA methylation of promoters	Stress-responsive and metabolic genes	Stress (drought)	Transcriptional repression modulating sugar metabolism genes	[105–107]
**Cucumber (*Cucumis sativus*) **	lncRNA–miRNA–mRNA (ceRNA network)	Sugar-related stress response genes	Heat stress and sugar stress	Enhances thermotolerance and sugar stress response via RNA scaffolding	[108]

## Sugar signaling along ripening: from accumulation to gene control

During ripening, assimilates accumulate and relocate, which reshapes transcriptional programs that govern pigment biosynthesis, cell-wall remodeling, and volatile compounds production, thereby determining fruit quality [109]. The content, identity, and ratio of specific sugars, including sucrose, glucose, and fructose, modulate ripening-associated genes, often via epigenetic mechanisms that adjust chromatin accessibility and promoter activity [110]. Thus, sugar signaling is both an upstream trigger and a downstream readout of ripening state, creating feedback loops between metabolism and gene regulation.

### Sugar-responsive epigenetic regulation of ripening pigments

Changes in determination, including anthocyanin and carotenoid accumulation alongside chlorophyll degradation, are hallmark ripening events sensitive to sugar supply. Sugar availability positively correlates with anthocyanin deposition in grapes, strawberries, blueberries, and cherries; sugar limitation delays coloration [111–113]. Carotenoid accumulation similarly benefits from high sugar status in tomato and other species [114].

Epigenetically, HDACs and DNA methylation constrain transcription in the pigment pathway [115]. In tomato, SlHDA1 and SlHDA3 suppress carotenoid accumulation and delay ripening [110]. In grape, VvHDAC19 recruits the ethylene response factor (ERF) VvERF4 to the *VvMYB5a* promoter, reducing H3/H4 acetylation and repressing anthocyanin biosynthesis, thereby delaying coloration [96]. Ripening-associated DNA hypermethylation perturbs the expression of several genes involved in orange ripening [103, 104]. During berry-skin coloration, induction of the DNA methyltransferase genes *VvMET2b/3* and *VvCMT2b/3* coincides with methylome remodeling across flavonoid genes, promoting anthocyanin accumulation at defined post-flowering stages [116]. Furthermore, increased global methylation levels occur in sweet orange during ripening, and extensive demethylation were reported in promoter regions of ripening regulators RIPENING INHIBITOR (*RIN*) and FRUITFULL 1 (*FUL1*) [103]. Moreover, in the naturally occurring tomato mutant *Colorless non-ripening* (*Cnr*), the *LeSPL-CNR* gene promoter exhibits aberrant hypermethylation, resulting in transcriptional silencing and failure of normal fruit ripening [117].

### Sugar metabolism in fruit softening: chromatin regulation of cell-wall enzymes and ethylene - ABA crosstalk

Softening requires coordinated activation of cell-wall hydrolases, including polygalacturonase, pectin methylesterase, β-galactosidase, and cellulase, also known as endo-1,4-β-glucanase [118]. In tomato, RNAi silencing of the DNA demethylase *SlDML2* hypermethylates promoters of softening genes, repressing their transcription and delaying softening [119] (Fig. 2D). HDACs exert bidirectional control: suppressing *SlHDA1/SlHDA3* elevates expression of cell-wall metabolism genes, such as *SlHEX, SlMAN, SlTBG4, SlXTH5* and *SlXYL*, and accelerates softening, whereas down-regulating *SlHDT3* reduces their expression and prolongs shelf life [110, 115]. In grape, DNA methylation of *VvPE*, a gene encoding a key pectin-degrading enzyme, critically modulates softening kinetics [120]. Hormone pathways interlock with sugar signaling to coordinate ripening [44, 116]. In climacteric fruits, such as tomato, ethylene enhances sucrose accumulation and ripening progression [121]. ERF transcription factors integrate ethylene with chromatin remodeling: tomato SlHDA1/SlHDA3 complex with SlERF.F12 to repress ethylene biosynthetic genes, including SlACS2/SlACS4, by reducing H3K9ac/H3K27ac, delaying ripening [122, 123].

In grape, a non-climacteric fruit, sugar rise accompanies ABA increases at ripening onset [124]. Exogenous ABA reshapes the methylome and modulates ripening and stress-responsive genes, underscoring hormone–epigenome rewiring downstream of metabolic status [116]. In strawberry, methylome and small-RNA profiling reveals ripening-associated DNA hypomethylation driven mainly by downregulation of RdDM components and diminished 24-nt siRNAs; 5-azacytidine treatment or silencing the RdDM effector *FvAGO4* triggers early ripening [88]. Light integrates into this circuit: dark treatment suppresses anthocyanin and sugar accumulation, while blue-light signaling through FaCRY1 diminishes methylation at the *FaCYP707A4* promoter by regulating the RdDM component *FaAGO4*. This leads to lowering ABA catabolism, increasing ABA levels, and promoting sugar accumulation and softening [125]. Under dormancy and stress, coordinated shifts in methylation and hormone content further support an integrated epigenetic response [89].

## Epigenetic regulation in sugar crops: a horticultural perspective

Sugar crops, owing to their high sugar-accumulating ability, are worthy examples to be explored for epigenetic profiles that dynamically regulate agronomic traits, the sugar biomass paradigm, and stress adaptation. Fruit sweetness is largely determined by the balance between soluble sugars (e.g. sucrose, glucose, fructose) and organic acids, commonly expressed as the sugar–acid ratio, extensively shaped by epigenetic regulation.

In sugarcane, sucrose accumulation is governed by tissue-specific epigenetic reprogramming; for example, DNA methylation valleys (DMVs) maintain stable, hypomethylated states at the promoters of core sucrose genes, while dynamic CHH-context DMRs act as control points for photosynthesis and transport [36]. Active demethylation in parenchyma tissues correlates with elevated expression of sucrose synthase and transporters, facilitating sink strength [36, 126]. Post-transcriptional control further refines sink strength, as miRNAs, including miR172-y and miR164-x, target transcripts associated with invertases and hexose transport, tuning sucrose cleavage and hexose retrieval in sink tissues. Sugar beet illustrates stress-responsive epigenetic plasticity. Cold exposure induces genome-wide hypomethylation through the repression of *CHROMOMETHYLASE 2* (*CMT2*) and *REPRESSOR OF SILENCING 1* (*ROS1*)*-*mediated demethylation, altering expression of vacuolar sucrose transporters, specifically *BvTST2.1* and *BvSUT4*, to optimize seasonal sucrose storage and remobilization [102, 127]. Under salinity, histone acetyltransferase BvHAC2 acetylates H3K18 residues, activating antioxidant pathways and osmoprotectant biosynthesis—a key mechanism underlying this crop’s exceptional tolerance [128, 129].

Key gaps limit the application of epigenetics in sugar crops: the stability of stress-induced marks across clonal generations is unclear [36, 130]; causal links between specific marks and traits require validation using targeted epigenome editing; and cross-talk among DNA methylation, histone modifications, and non-coding RNAs remains poorly resolved in complex genomes. Resolving these issues could enable nontransgenic improvement of sink strength, sugar storage, and abiotic stress tolerance.

## Epigenetic regulation of sugar signaling and sweetness formation in horticultural crops

### Chromatin accessibility and transcriptional networks controlling sugar–acid metabolism

High-resolution chromatin profiling is enabled to reveal how accessibility dynamics coordinate sugar-related gene expression programs during fruit development. In apple, integrative ATAC-seq and RNA-seq analyses across developmental stages identified stage-dependent chromatin accessibility changes that coincide with the activation of sugar-accumulation programs. Mechanistically, this fits the broader model that sugar flux rewires epigenetic capacity by shifting acetyl-CoA, NAD^+^, α-ketoglutarate (α-KG), and S-adenosylmethionine (SAM), which fuel histone acetylation, deacetylation, methylation, and demethylation. Concurrently, sugar-responsive TF circuits act as the gene-regulatory output; consistent with that, fruit-enriched DNA-binding with one finger (Dof) TFs were functionally shown through overexpression and virus-induced gene silencing (VIGS) in ‘Fuji’ fruit to regulate *MdBAM3* and *MdTST2*, which are responsible for quantitative changes in sugar concentration [131].

### DNA methylation as a stage-dependent switch in sugar signaling

DNA methylation, often site- and context-specific, influences the expression of carbohydrate-related genes. In apple, mature fruits exhibit higher overall methylation, with changes associated with ripening, sugar metabolism, and hormone signaling. Certain methylation patterns, like increased promoter CHH or reduced CG methylation, are associated with higher gene expression. Reduced methylation in non-promoter regions also aligns with upregulation. These findings show that the effect of methylation on transcription depends on its context and location, adding complexity to gene regulation during ripening [51]. Cold stress triggers a coordinated transcriptional and epigenetic reprogramming of sugar metabolism in potato, primarily driving cold-induced sweetening (CIS) through the activation of the vacuolar invertase gene *StINV1* and related genes. A cis-regulatory intronic enhancer within *StINV1* was shown to be essential for its cold-responsive expression, mediating a 3- to 4-fold transcriptional increase via temperature-sensitive elements and binding sites for cold-responsive transcription factors [132]. Complementing this, genome-wide epigenomic profiling revealed that cold stress enhances chromatin accessibility and deposits bivalent histone marks, specifically H3K4me3 and H3K27me3, at over a thousand cold-responsive genes, including those involved in carbohydrate metabolism. These findings suggest a multilayered regulatory model wherein cis-enhancers, chromatin remodeling, and histone modifications converge to fine-tune sugar gene expression under cold conditions, highlighting the epigenetic plasticity underlying CIS in potato [133].

### Sugar metabolism in sweet and sour fruits

The stoichiometry of ‘sweetness and sourness’ is viewed as an epigenetically catered organization that runs in parallel with sugar status regulation. A comparative study identified the vacuolar proton-pump component *PH5*, which is tied to citric acid accumulation, as a causal link between the acidic ‘Eureka’ and the nearly non-acidic sweet lemon in a haplotype-resolved genome. The *PH5* promoter is highly methylated in sweet lemon but not in Eureka, and experimental demethylation of the *PH5* promoter increases citric acid content, directly connecting DNA methylation state to the acid phenotype [134]. During the developmental phases, lemon methylome profiling across fruit stages depicts DNA methylation remodeling and reports that CHH-hypermethylated DMRs are strongly associated with expression of genes important for citric-acid synthesis and accumulation, such as phosphoenolpyruvate carboxykinase (*ClPEPCK*). This supports the idea that methylation is not merely correlative but a coordinating layer over the citrate network during ripening [135]. Elevated H3/H4 histone acetylation at the *PuWRKY31* promoter enhances *PuWRKY31* transcription in a high-sucrose pear bud sport, highlighting an epigenetic mechanism underlying fruit sugar variation. The PuWRKY31 protein directly activates the sucrose transporter gene *PuSWEET15*, promoting sucrose accumulation during fruit development and linking chromatin state to sugar transport/metabolism in pear fruit (Fig. 2C and E) [136]. In the chromosome-scale *Cucumis melo* ssp. *agrestis* genome, the authors explicitly link sucrose accumulation to DNA methylation-associated expression differences, reporting that transcriptome + DNA-methylation pattern associations support epigenetic regulation of sucrose accumulation during fruit development, alongside mapping candidate sucrose loci, such as alkaline/neutral invertase *CINV*, sucrose-phosphatase 2, and α/β-galactosidases [137]. Functionally, later work in melon pinpoints a plausible ‘switch’: *CmROS1*, a DNA demethylase gene inside the ripening quantitative trait locus (QTL) *ETHQV8.1*, where CRISPR knockouts plus BS-seq show altered methylation dynamics and methylation changes in promoter regions of ethylene pathway genes, including *ACS1*, *ACO1*, and *ETR1*, as well as master ripening transcription factor genes, such as *NAC-NOR*, *RIN*, and *CNR*, ultimately, shifting ripening timing [138]. Finally, post-transcriptional epigenetic layers also plug into this timing control: melon ripening stage miRNA/degradome work identifies differentially expressed miRNAs with validated targets and shows that manipulating a ripening-related miRNA module can delay ripening, consistent with epigenetic regulation acting upstream of the metabolic shift that culminates in high sucrose [139].

Taken together, these studies indicate that epigenetic regulation is a conserved feature of sugar signaling, yet it operates through species, tissue, and stage-specific architectures. Several recurring principles emerge: (i) epigenetic states frequently converge on hub processes, including sucrose-starch interconversion, invertase-mediated sucrose cleavage, sugar transport, and hormone–sugar crosstalk; (ii) regulatory effects are often context-dependent, varying between promoters and gene bodies, among CG and CHH contexts, or between accessibility and methylation states; and (iii) environmental inputs, such as cold storage, can induce chromatin remodeling that redirects carbohydrate flux.

## Epigenetic applications in crop technologies for sweetness and stress resilience

### Exploiting transgenerational epigenetic inheritance of sugar-related traits

**Figure 4:**
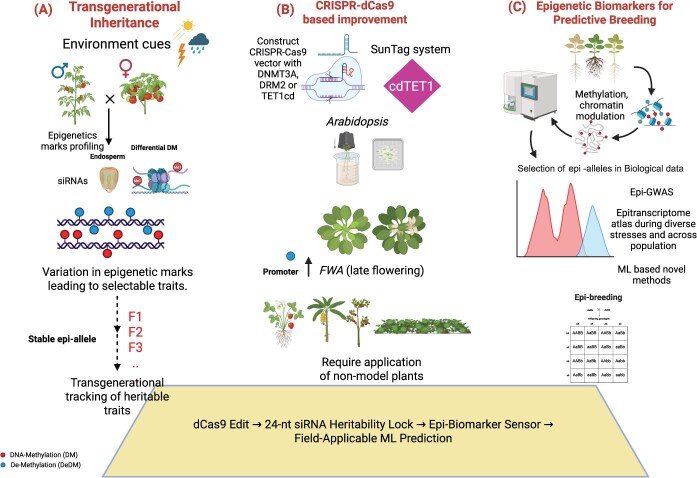
Epigenetic innovations in crop improvement. Panel (A) showcases CRISPR-dCas9 epigenome editing approaches (e.g. SunTag-TET1cd, DRM2 fusions) to precisely modulate sugar metabolism genes via targeted methylation/demethylation, enabling trait engineering without DNA changes. Panel (B) highlights transgenerational epigenetic inheritance, where hybrid vigor and epi-QTLs, such as *CmWIP1*, *EgDEF1*, exploit heritable methylation shifts driven by TEs and RdDM, to control ripening, yield, and stress resilience. Panel (C) illustrates epigenetic biomarkers for sugar signaling (e.g. *FLC* demethylation in sugar beet) and ML to accelerate breeding for sucrose content and adaptive traits.

Transgenerational epigenetic inheritance (TEI) offers a promising approach for enhancing crop traits, particularly when integrated with heterosis, also known as hybrid vigor, and metabolic pathway engineering (Fig. 4A). A central idea is that ‘genome shock’ triggers TEs and remodels epigenetic reprogramming. In some cases, these induced epigenetic states form stable epialleles. For example, heterosis in sugarcane (*Saccharum* spp.) and sugar beet (*Beta vulgaris*) correlates with heritable DNA methylation patterns at heterochromatic knobs and TE-rich regions, suggesting a role for epigenetic regulation in hybrid vigor. Inter-specific hybridization often induces trans-acting methylation (TAM) and trans-acting demethylation (TAdM) events, generating epialleles stabilized by siRNAs and the RdDM pathway. In maize × teosinte hybrids, hybridization-induced paramutation creates heritable trans-acting epialleles that persist over six backcross generations, mediated by 24-nt siRNAs via canonical RdDM [9]. Crucially, these epialleles modulate the expression of genes involved in carbohydrate metabolism and photosynthesis, driving phenotypic variations in seedling biomass and environmental adaptation. Similarly, maternal 24-nt siRNAs produced by RNA polymerase IV (Pol IV), encoded by the NRPD1a gene, silence TEs and genes, such as AGAMOUS-LIKE (*AGL*) genes in hybrid endosperm, regulating seed development; modulating siRNA dosage could improve seed viability in wide crosses [140].

Epigenetic quantitative trait loci (epi-QTLs) powerfully demonstrate the agronomic significance of TE insertions by linking epigenetic variation to key crop traits. In tomato, methylation-sensitive epi-QTLs affecting *SBP-box* genes like *Cnr* control fruit maturation through promoter hypermethylation, directly impacting ripening and shelf life [141]. Similarly, in melon, the methylation status of *CmWIP1* acts as an epi-QTL regulating gynoecy, or female flower development, a critical determinant of yield [142, 143]. Rice architecture is shaped by epi-QTLs, such as those governing *OsDWARF1*, where differential methylation creates epialleles that alter gibberellin signaling, thereby modifying plant height and tillering traits vital for lodging resistance and harvest efficiency [144]. Perhaps most strikingly, hypomethylation of a *LINE* retrotransposon near the *EgDEF1* gene in oil palm creates a stably inherited epi-QTL responsible for the ‘mantled’ fruit phenotype, which causes abnormal development and severe commercial losses [145]. Collectively, these examples underscore epi-QTLs as major drivers of agriculturally critical variation, highlighting TEI-mediated epigenetic regulation as both a challenge, such as somaclonal variation, and a potential tool for crop improvement.

These mechanisms extend to metabolic traits. For instance, epi-QTLs govern vitamin E accumulation in tomato [138]. Critically, epigenetic variations often target metabolic genes, suggesting potential for engineering sugar accumulation pathways. Woodland strawberry additionally shows that stress-induced DNA methylation marks, known as epimutations, can be stably transmitted through ≥3 asexual generations, highlighting TEI in clonally propagated fruit crops [146]. Sugar-linked epigenetic variation is detectable in apple, where cultivar differences in soluble sugars coincide with coordinated differences in the expression of sugar metabolism and transport genes, along with specific promoter and gene-body methylation patterns. Similarly, in grape berries, methylation-responsive genes are enriched for ‘fructose and mannose metabolism’ and ‘glycolysis/gluconeogenesis,’ including a promoter-hypermethylated, downregulated phosphofructokinase gene consistent with hexose accumulation during ripening [147, 148]. These crop-specific observations converge on conserved sugar-hubs, including the HXK, SnRK1, and TOR proteins, alongside T6P signaling, and hormone cross-talk described for fleshy fruits; for example, *FaSnRK1α* manipulation in strawberry coordinately rewires sucrose metabolism and sucrose transporter expression to alter fruit sucrose content [149].

The stability of epialleles hinges on 24-nt siRNAs and RdDM, particularly in hybrid seed development [150, 151]. Notably, highly heterotic maize and sugarcane hybrids exhibit demethylation at carbohydrate metabolism loci, correlating with vigor. Paramutation events, such as methylation of an *FLC*-like gene in sugar beet, also influence flowering [152], though outcomes remain context-dependent. Predictive frameworks are needed to distinguish functional epialleles from bystander effects. Epigenetic recombinant inbred lines (epiRILs) enable the dissection of epigenetic versus genetic contributions to traits. Integrating methylome data with ATAC-seq chromatin accessibility and ChIP-seq histone modification profiles will elucidate multi-layered heterosis mechanisms. We propose developing hybrid epigenome maps tracking methylation, histone marks, and siRNAs across F₁, F₂, and backcross generations to decode the epigenetic basis of hybrid vigor.

### Epigenetic engineering for sweetness

Epigenome editing via CRISPR-dCas9 fusions provides a transformative route to modulate sugar accumulation in horticultural and industrial crops without altering genomic sequences (Fig. 4B). Fusion of dCas9 with DNA methyltransferases, for example, DNMT3A, DRM2 or demethylases TET1cd, may benefit precise modification of promoter methylation at key sugar metabolism genes, such as SUCROSE SYNTHASE (*SUS*), INVERTASE (*INV*), and SWEET family transporters. For instance, the fusion of the dCas9 endonuclease with the catalytic domain of DNA methyltransferase DNMT3A, linked by a flexible Gly4Ser linker, created a tool for targeted CpG methylation [153]. The SunTag system offers an efficient method to tether proteins to dCas9, using tandem GCN4 peptide repeats fused to dCas9 and a single-chain GCN4 antibody with epigenetic effectors [154, 155]. This approach was tested with the DNA demethylase cdTET1 in *Arabidopsis*, showing high efficiency of demethylation at the targeted *FWA* locus [156]. Furthermore, tethering CRISPR–dCas9-SunTag to DRM2, a *de novo* DNA methyltransferase, was also shown to work efficiently at the *FWA* locus in *Arabidopsis* [157]. CRISPR-based genome editing is being used both to directly rewire sugar accumulation and to interrogate epigenetic regulators that gate ripening-linked metabolic shifts. In melon, CRISPR knockouts of two *ETHQV8.1* candidates— *CTR1-like* (a negative regulator of ethylene signaling) and *CmROS1* (a DNA demethylase orthologous to tomato *DML2* )— shifted climacteric behavior toward an earlier ethylene profile. Furthermore, loss of *CmROS1* remodeled cytosine methylation at promoters of key ripening genes (*ACS1*, *ACO1*, and *ETR1*) and master regulators (*NAC*-*NOR*, *RIN*, and *CNR*), connecting active DNA demethylation to ripening control [138]. In tomato, *SlDML2* loss-of-function mutants fail to ripen, revealing that DNA demethylation is required not only to activate ripening programs for pigment, flavor, and ethylene pathways but also to enable repression of large gene sets related to photosynthesis and cell-wall biosynthesis, highlighting coordinated ‘on/off’ control during ripening transitions [158]. Complementing these regulatory insights, editing sugar ‘hardware’ has produced measurable sweetness gains: disruption of the cell-wall invertase inhibitor *SlINVINH1* increased SSC and elevated fructose/glucose levels by ~29% to 36% without decreasing fruit weight in selected lines [159]. More recently, two tomato calcium-dependent protein kinases, SlCDPK26 and SlCDPK27, were identified as ‘sugar brakes’; gene-edited knockouts increased glucose and fructose by up to 30% and improved perceived sweetness without yield penalty, mechanistically linked to sucrose synthase stability [160]. Beyond tomato, multiplex CRISPR editing of grape tonoplast monosaccharide transporters, specifically *TMT1* and *TMT2*, reduced sugar levels, providing a tractable route to dissect vacuolar sugar sequestration in berries [161]. In parallel, methylome work in non-climacteric strawberry shows ripening-associated DNA hypomethylation tied to downregulation of RdDM, suggesting conserved epigenetic reprogramming logic across fruit types [88]. Finally, emerging dCas9-based epigenome editing, such as SunTag systems for locus-specific methylation and activation in plants, offers a path to modulate ripening/sweetness loci without changing coding sequences - an attractive next step for precision quality breeding [157].

## **Epigenetic** b**iomarkers for** a**ccelerated** b**reeding**

Robust epigenetic biomarkers offer significant potential to accelerate selection for complex traits such as sugar accumulation, flowering time, and stress resilience (Fig. 4C). Unlike genetic markers, epigenetic signatures such as DNA methylation patterns and histone modifications, capture both heritable variation and environmental plasticity. In sugar beet (*Beta vulgaris*), demethylation of *FLC*-like intronic regions predicts early flowering at the seedling stage [152]. Genome-wide methylation profiling in tomato and grapevine has similarly identified conserved DMRs associated with soluble solids content and ripening kinetics. ML approaches—particularly convolutional neural networks (CNNs) trained on BS-seq data—now enable accurate prediction of sucrose levels (achieving *R*^2^ > 0.75) and stress tolerance [102, 162]. However, translational applications remain limited by the context dependency of epigenetic states. We propose an epigenome-wide association study (Epi-GWAS) and ML pipeline combining epigenomic data with deep learning for cross-environment prediction. This framework could enable early selection of epialleles linked to sugar metabolism, particularly in resource-limited crops with costly phenotyping. Once validated across environments and genetic backgrounds, such DMRs can be converted into scalable assays, including targeted bisulfite and amplicon tests or MSAP-derived screens, to accelerate early-generation selection for high-sugar lines.

## Future research imperatives

This review underscores the pivotal role of epigenetic regulation in developmental phenology and plasticity. The integration of sugar metabolism with epigenetic mechanisms reveals complex regulatory networks that modulate phenotypic plasticity, influencing critical traits such as fruit quality, yield, and stress tolerance. The path forward in plant epigenetics demands a tripartite focus on resolving persistent mechanistic ambiguities, pioneering technologies, and accelerating translational applications. Future work should first prioritize converting current association-heavy maps into causal rules that explain how sugar status is relayed into chromatin change. This means experimentally linking sugar-sensing hubs, including HXK1, TOR, SnRK1, and T6P, to locus-specific epigenetic outcomes by combining targeted perturbations with direct tracking of DNA methylation, histone states, accessibility, and transcription at defined candidate loci, rather than relying on endpoint comparisons alone. A second imperative is to shift from single-timepoint designs to dense, time-resolved trajectories under controlled sugar inputs and relevant developmental or stress transitions. This review highlights that static interactions or correlational studies for histone marks can overlook combinatorial dynamics, feedback loops, and the emergence of regulatory ‘memory.’ Closely related, mechanistic studies should explain recruitment specificity: how sugar perception converges with chromatin regulators, including Polycomb-related routes, and how concurrent cues, such as light, ABA, ethylene, temperature, and stress, are integrated at shared loci during ripening and acclimation. Concurrently, integrating spatiotemporal dynamic epigenetic datasets with ML-driven models will transform predictive capacity, linking epigenetic landscapes to traits like sucrose yield or ripening in diverse environments. Translationally, precision epigenome editing must evolve from proof-of-concept to field-ready solutions. This entails engineering tissue-specific dCas9 effectors, such as SunTag-DRM2 and TET1 fusions, to stably reprogram DNA methylation in DMVs controlling genes encoding *SWEET* transporters or cell-wall hydrolases, while systematically tracking heritability in perennial polyploids over vegetative generations. Parallel efforts should establish epigenetic breeding pipelines anchored by environmentally robust biomarkers through epi-QTL mapping in staple crops and deploy convolutional neural networks to predict complex traits from seedling epigenomes. Ultimately, closing the loop from discovery to application requires the field validation of epigenetically edited lines, the exploitation of TEI in synthetic ‘epi-hybrids’, and the development of epigenetic priming strategies to enhance postharvest resilience, thereby positioning epigenetics as a cornerstone of climate-smart agriculture.
